# Fiske Fund Prize Essays

**Published:** 1860-11

**Authors:** 


FISKE FUND PRIZE ESSAYS.—The Trustees of the Fiske Fund announced at the meeting of the Rhode Island Medical Society, held July eleventh of this year, that premiums of one hundred dollars each had been awarded to Dr. W. W. Morland, of Boston, for a dissertation on Uræmia and its Morbid Effects; to Dr. D. W. Slade, of Boston, for his essay on Diphtheria.

The following are the subjects for the prizes for 1861: 1. Aneurism: its Varieties and their Appropriate Treatment. 2. Ozone: its Relations to Health and Disease. Dissertations on these subjects, marked by a motto, and accompanied by a sealed packet containing the same motto and the writer's name and residence, should be sent to Dr. S. A. Arnold, Providence, Rhode Island, on or before the 1st of May, 1861.

